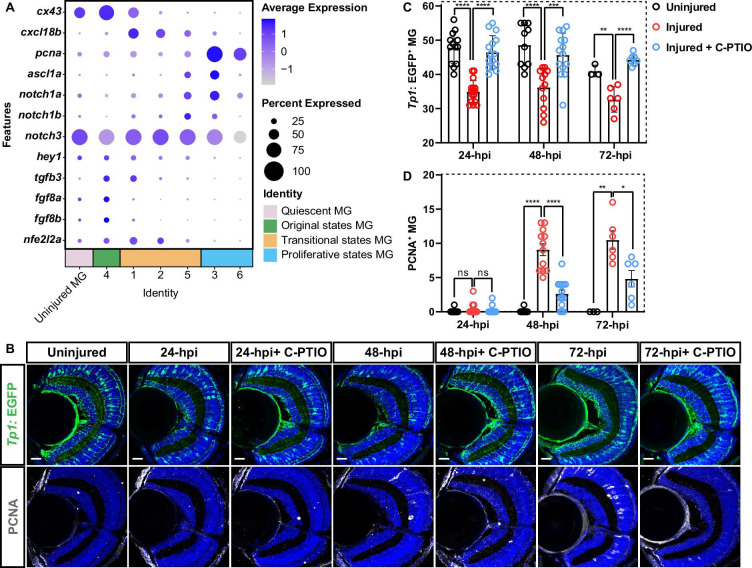# Correction: *cxcl18b*-defined transitional state-specific nitric oxide drives injury-induced Müller glia cell-cycle re-entry in the zebrafish retina

**DOI:** 10.7554/eLife.112806

**Published:** 2026-08-03

**Authors:** Aojun Ye, Shuguang Yu, Meng Du, Dongming Zhou, Jie He, Chang Chen

**Keywords:** Zebrafish

Ye A, Yu S, Du M, Zhou D, He J, Chen C. 2026. cxcl18b-defined transitional state-specific nitric oxide drives injury-induced Müller glia cell-cycle re-entry in the zebrafish retina. *eLife* **14**:RP106274. doi: 10.7554/eLife.106274.

Published 21 January 2026

Following an internal review of the published article, we wish to correct several inadvertent errors affecting Figure 4, Figure 7B, Figure 1—figure supplement 1A, Figure 2—figure supplement 1H, and Figure 4—figure supplement 1D.

In Figure 4B, the quantification originally included data from only 11 wild-type (WT) control samples. We have now updated this panel to include all available WT control cohorts generated under identical injury conditions and analyzed using the same criteria (total n=22). Re-analysis with the complete dataset confirms that all statistical outcomes and biological conclusions remain unchanged.

During figure assembly, several representative control images were inadvertently duplicated across panels. The retinal injury (72 hpi), NOS knockout, and NOS inhibitor experiments were performed in parallel within the same retinal injury paradigm and shared common WT or scrambled control cohorts at the 72-hpi endpoint. During figure assembly, representative images from these shared control cohorts were inadvertently reused across multiple related figures and experimental comparisons. As a result, the same image was displayed in different figure panels. Specifically, the WT retinal injury time-course image shown in Figure 1—figure supplement 1A (72-hpi) was reused in Figure 4A (WT), Figure 4D (Uninjected), Figure 2—figure supplement 1H (WT), and as different channel views in Figure 4—figure supplement 1D. Additionally, the scrambled control image shown in Figure 2—figure supplement 1H was reused in Figure 4A (Scrambled). These panels have now been replaced with independent representative images from the corresponding WT or scrambled control cohorts. The revised Figure 4A, Figure 4D, Figure 2—figure supplement 1H, and Figure 4—figure supplement 1D accordingly display new, distinct control images, and Figure 4B now reflects the complete WT dataset.

In Figure 7B, the *Tp1:* EGFP panels for the “72-hpi” and “72-hpi+C-PTIO” conditions were inadvertently inverted during assembly. The error occurred during the arrangement and annotation of the representative image panels in the course of figure preparation, which led to the misplacement of two representative images. After reviewing the original image files, we confirmed that the underlying raw data and all quantitative analyses remain correct, and that the discrepancy was restricted to panel positioning at the figure assembly stage.

The corrected Figure 7B now accurately presents the correct images for each condition.

All original raw imaging files, experimental records, and quantification files remain fully available and traceable. These corrections do not alter the statistical analyses, data interpretation, or conclusions of the study. We sincerely apologize to the readers for these errors and any confusion they may have caused.

The corrected figures and corresponding legends are provided below.

1. Figure 1—figure supplement 1

The corrected Figure 1—figure supplement 1A with updated panel 72-hpi is shown here:

**Figure 1:**
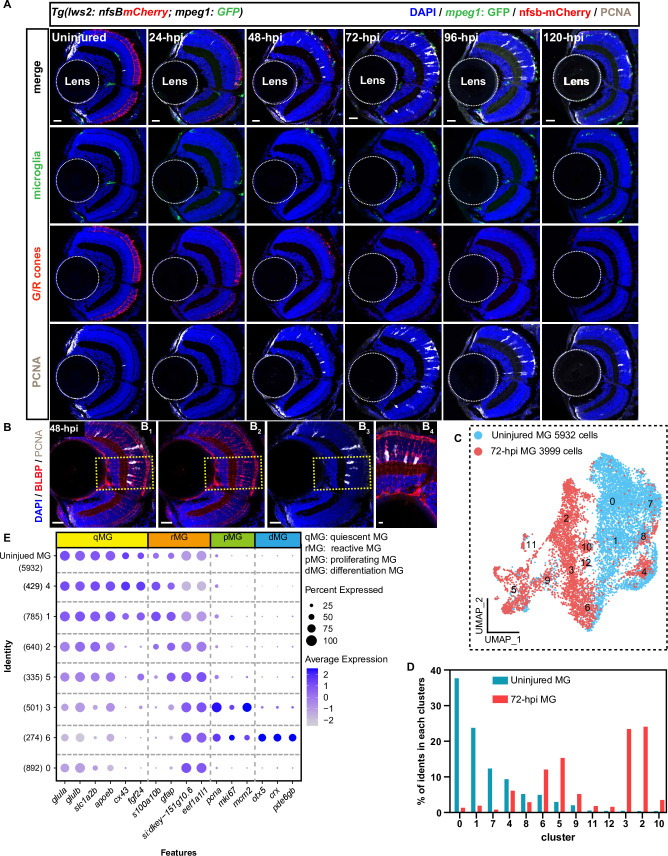


The originally published Figure 1—figure supplement 1 is shown here:

**Figure 2:**
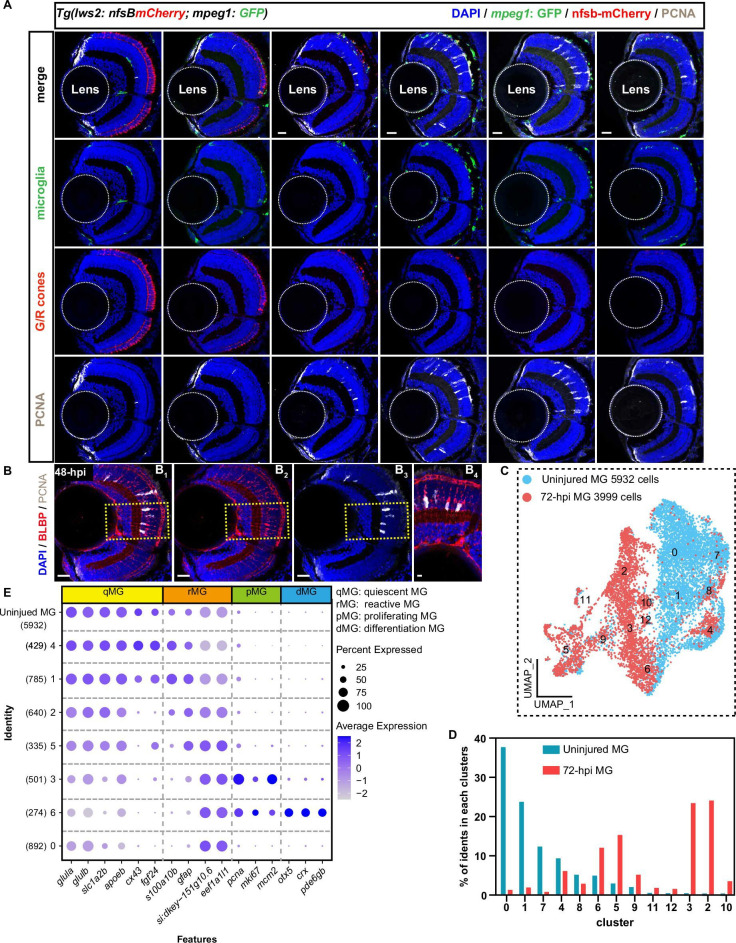


2. Figure 2—figure supplement 1

The corrected Figure 2—figure supplement 1H with updated WT panel is shown here:

**Figure 3:**
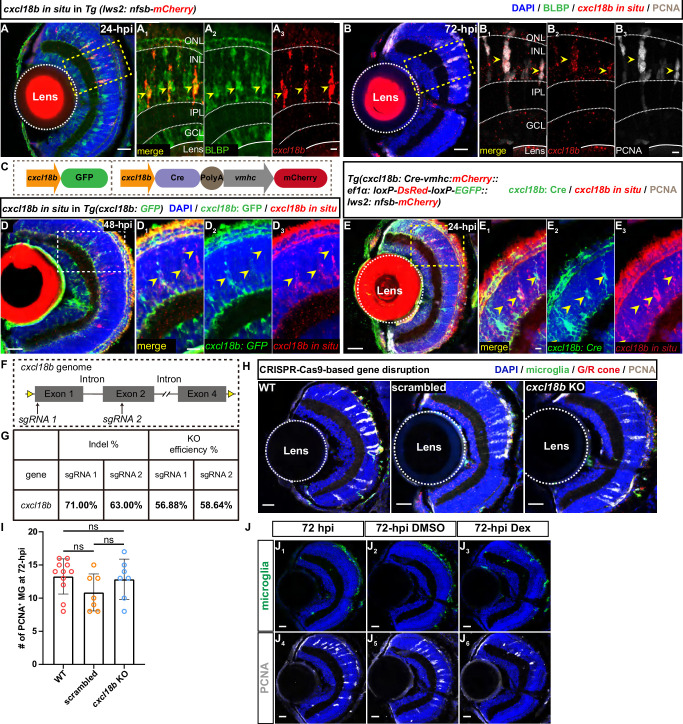


The originally published Figure 2—figure supplement 1 is shown here:

**Figure 4:**
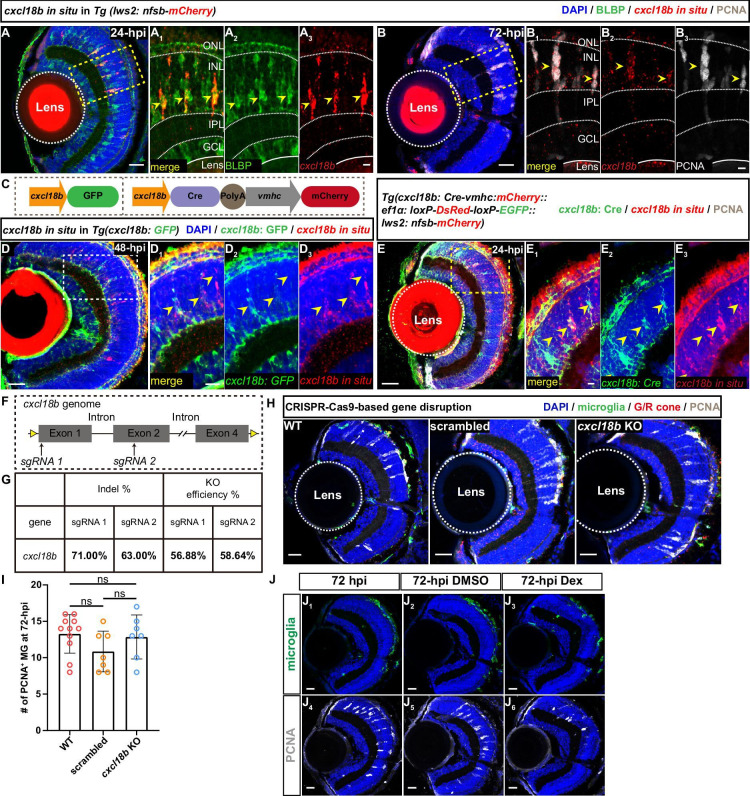


Corrected text in Figure 2—figure supplement 1 legend:

Gene disruption of *cxcl18b* does not reduce Müller glia (MG) proliferation.

Original text in Figure 2—figure supplement 1 legend:

Gene description of cxcl18b does not reduce Müller glia (MG) proliferation.

3. Figure 4

The corrected Figure 4 with updated WT and scrambled panels in Figure 4A, updated WT quantification in Figure 4B, and updated Uninjected panel in Figure 4D is shown here:

**Figure 5:**
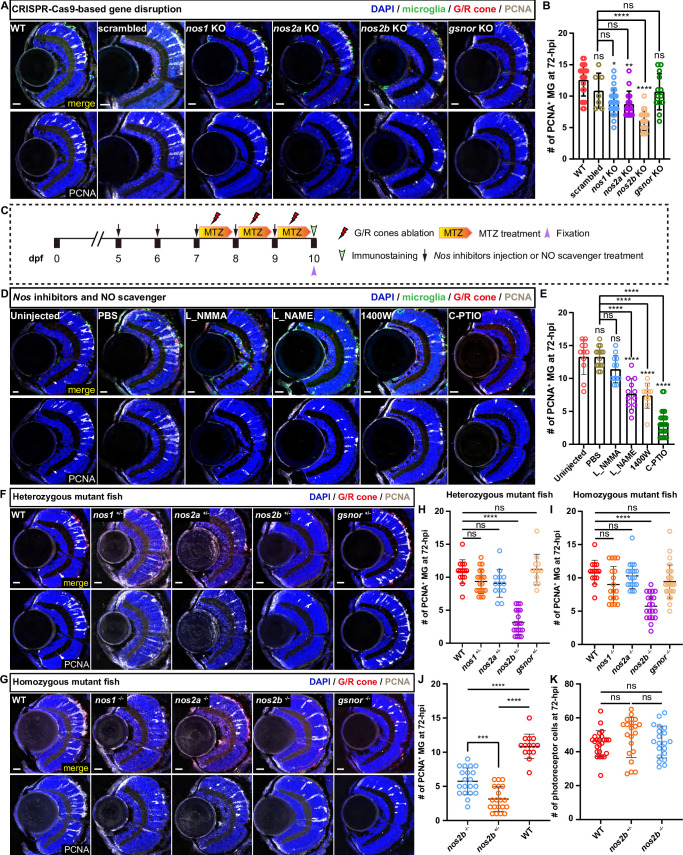


The originally published Figure 4 is shown here:

**Figure 6:**
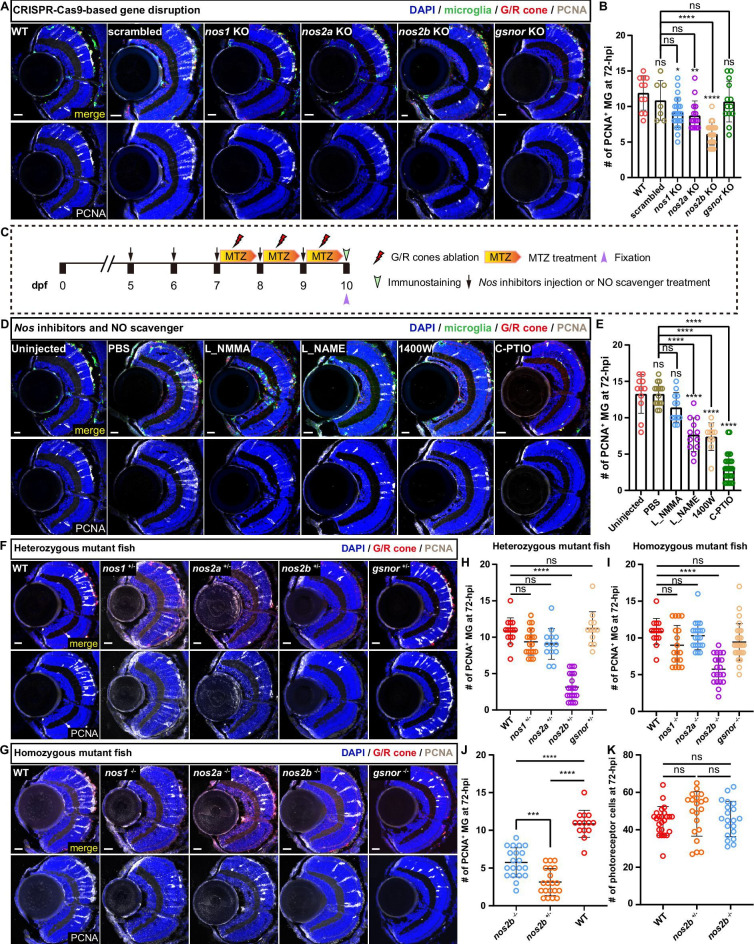


Corrected text in Figure 4B legend:

(**B**) Quantitative analysis of the number of proliferative MG (PCNA^+^) at 72 hpi in (**A**). In total, we collected **22** retinas for WT (n=**22**), *scrambled* sgRNA-injection (n=7), *nos1* sgRNA-injection (n=23), *nos2a* sgRNA-injection (n=15), *nos2b* sgRNA-injection (n=22), and *gsnor* sgRNA-injection (n=13) (mean ± SEM; ****p<0.0001, **p<0.01, *p<0.05, ns, p>0.05; one-way ANOVA followed by Tukey’s HSD test).

Original text in Figure 4B legend:

(**B**) Quantitative analysis of the number of proliferative MG (PCNA^+^) at 72 hpi in (**A**). In total, we collected **11** retinas for WT (n=**11**), *scramble* sgRNA-injection (n=7), *nos1* sgRNA-injection (n=23), *nos2a* sgRNA-injection (n=15), *nos2b* sgRNA-injection (n=22), and *gsnor* sgRNA-injection (n=13) (mean ± SEM; ****p<0.0001, **p<0.01, *p<0.05, ns, p>0.05; one-way ANOVA followed by Tukey’s HSD test).

4. Figure 4—figure supplement 1

The corrected Figure 4—figure supplement 1 with updated panel Figure 4—figure supplement 1D-WT and scrambled is shown here:

**Figure 7:**
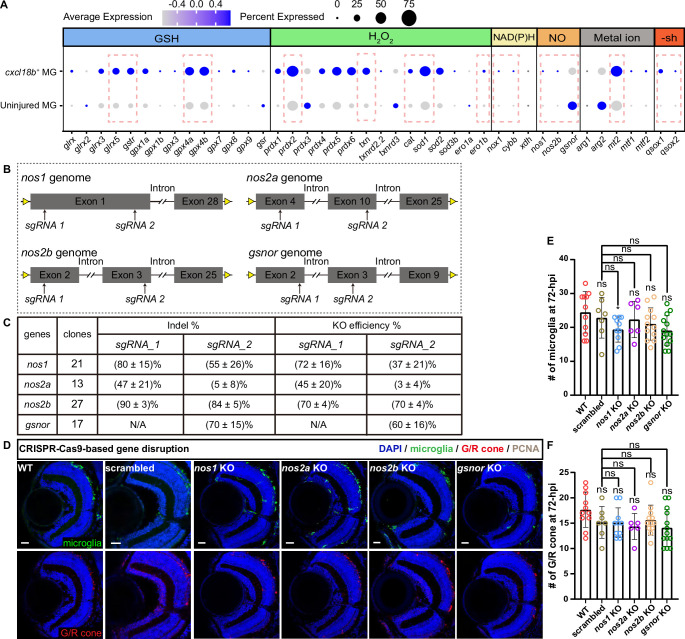


The originally published Figure 4—figure supplement 1 is shown here:

**Figure 8:**
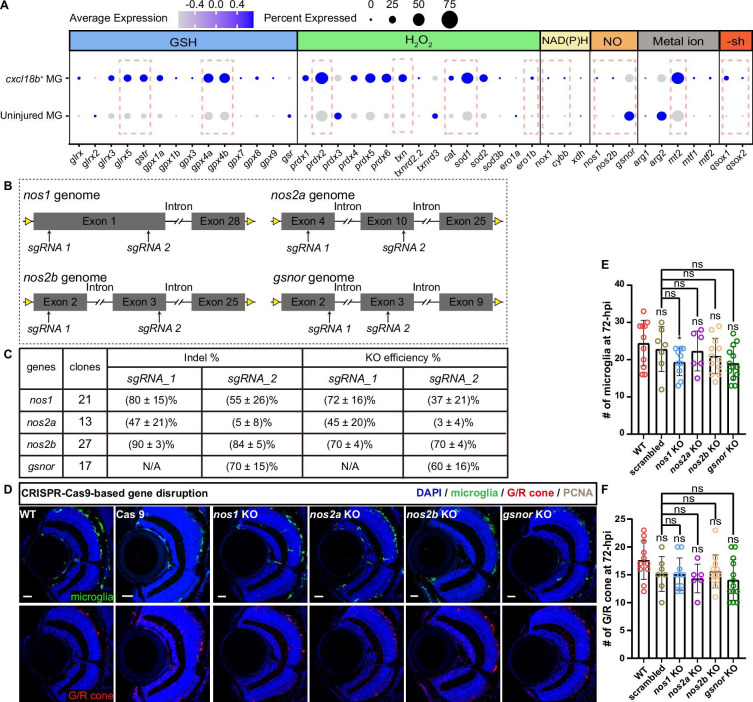


5. Figure 7

The corrected Figure 7 with updated panel Figure 7B-72-hpi and 72-hpi+C PTIO is shown here:

**Figure 9:**
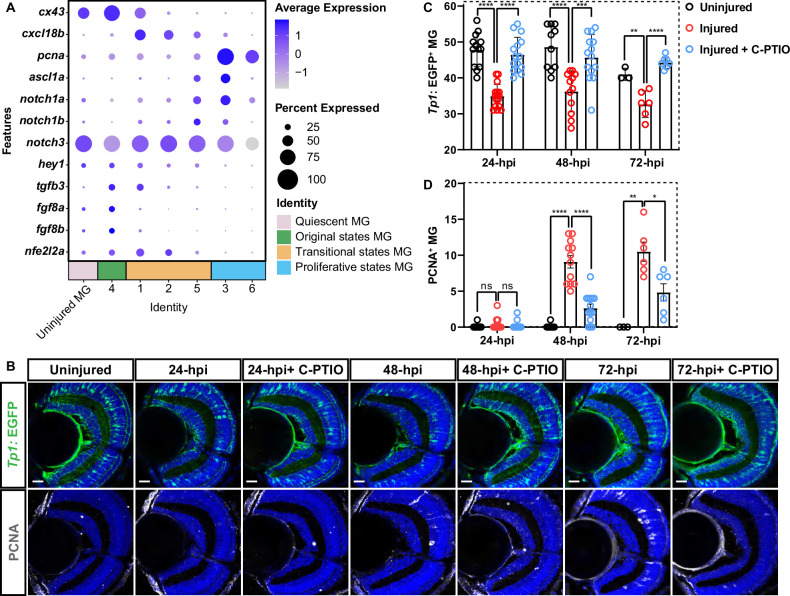


The originally published Figure 7 is shown here:

**Figure 10:**