# Supplementary material for: cxcl18b-defined transitional state-specific nitric oxide drives injury-induced Müller glia cell-cycle re-entry in the zebrafish retina
Source: eLife. 2026 Jan 21;14:RP106274. doi: 10.7554/eLife.106274 (PMC12823065; doi:10.7554/eLife.106274)
Supplement: Figure 6—figure supplement 1—source data 1. [file elife-106274-fig6-figsupp1-data1.zip › Figure 6-figure supplement 1_Source Data 1.pdf]

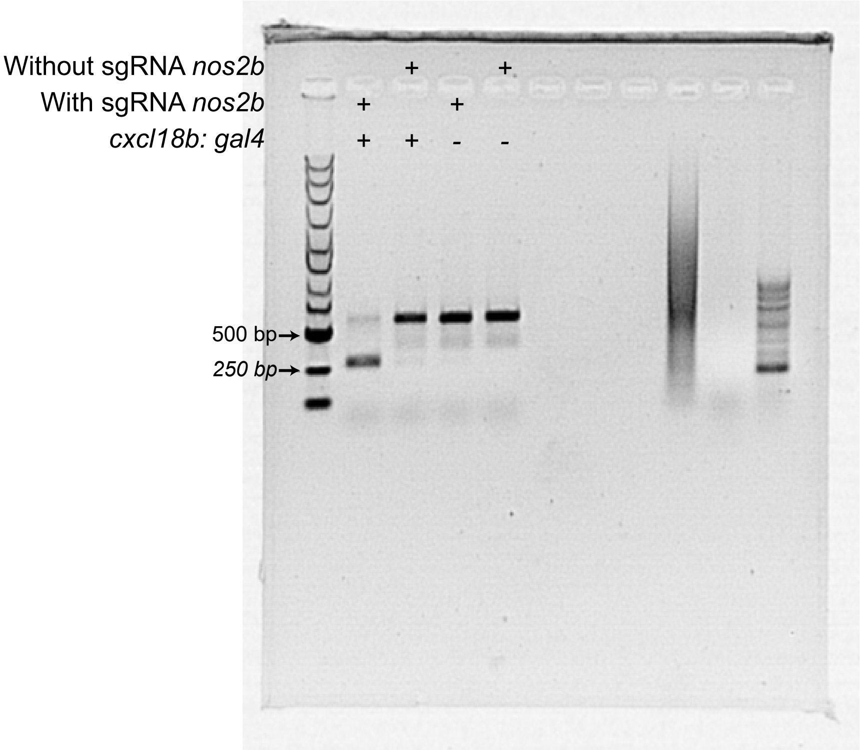

Figure 6–figure supplement 1, Source Data 1. Original gel image corresponding to Figure 6–figure supplement 1, panel C. PCR analysis of MG cell–specific gene knockout shows a truncated ***nos2b*** PCR product (~250 bp), indicative of gene disruption, only in MGs co-infected with ***cxcl18b:gal4–*** and ***nos2b*** sgRNA–expressing AAVs (lane 1). In contrast, dual-AAV infection with a scrambled sgRNA control (lane 2) or infection with either AAV alone (lanes 3–4) yields only the full-length amplicon (~500 bp), indicating that effective ***nos2b*** knockout in MG cells requires dual-AAV delivery of ***cxcl18b:gal4***–driven Cas9 and the targeting sgRNA.
