# Supplementary figures and images for: cxcl18b-defined transitional state-specific nitric oxide drives injury-induced Müller glia cell-cycle re-entry in the zebrafish retina
Source: eLife. 2026 Jan 21;14:RP106274. doi: 10.7554/eLife.106274 (PMC12823065; doi:10.7554/eLife.106274)

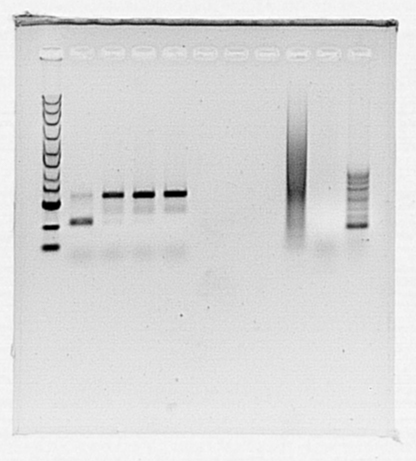

Supplement: Figure 6—figure supplement 1—source data 2. [file elife-106274-fig6-figsupp1-data2.zip › Figure 6-figure supplement 1_Source Data 2.jpg]
