# Supplementary material for: cxcl18b-defined transitional state-specific nitric oxide drives injury-induced Müller glia cell-cycle re-entry in the zebrafish retina
Source: eLife. 2026 Jan 21;14:RP106274. doi: 10.7554/eLife.106274 (PMC12823065; doi:10.7554/eLife.106274)
Supplement: Supplementary file 1. [file elife-106274-supp1.docx]

**Supplementary File 1a. sgRNA sequences and genotyping primers, related to STAR Methods.**

| **sgRNA targets sequences** | |
| --- | --- |
| **Gene symbol** | **Primer forward** |
| *nos1* sgRNA target-1 | TAATACGACTCACTATAGGCTCCCAGGAAAGAGTCGCGTTTTAGAGCTAGAAATAGC |
| *nos1* sgRNA target-2 | TAATACGACTCACTATAGGTCACCAGGCCTGTCAACCGTTTTAGAGCTAGAAATAGC |
| *nos2a* sgRNA target-1 | TAATACGACTCACTATAGGAGGAACTCGCTTTTGGGGGTTTTAGAGCTAGAAATAGC |
| *nos2a* sgRNA target-2 | TAATACGACTCACTATAGGTGGCTGGTGCCACCGATGGTTTTAGAGCTAGAAATAGC |
| *nos2b* sgRNA target-1 | TAATACGACTCACTATAGGAGTATTTCAGAAACACATGTTTTAGAGCTAGAAATAGC |
| *nos2b* sgRNA target-2 | TAATACGACTCACTATAGGATGTGTTACATCACAGAGGTTTTAGAGCTAGAAATAGC |
| *gsnor* sgRNA target-1 | TAATACGACTCACTATAGGCCCATGAAGTTCGAGTGAGTTTTAGAGCTAGAAATAGC |
| *gsnor* sgRNA target-2 | TAATACGACTCACTATAGGTCATCCTGGGTCATGAGGGTTTTAGAGCTAGAAATAGC |

**Supplementary File 1b. sgRNA sequences and genotyping primers, related to STAR Methods.**

| **Genotyping primers for gene disruption** | | |
| --- | --- | --- |
| **Gene symbol** | **Primer forward** | **Primer reverse** |
| *nos1*-sgTarget-1 | ATCGTCTCGGACCTCATCCG | ATAGGTTTTCTATTGCCCTGAGCTC |
| *nos1*-sgTarget-2 | ATGCGAACGATGCTCGC | ATCCTTCCTATCGATGACCCTCTGG |
| *nos2a*-sgTarget-1 | ATTACTGTTCTGTCGACTTGATAAACACG | ATACTTAAGATGAGTGCACAAGGCATG |
| *nos2a*-sgTarget-2 | ATTTAGCCACTCTGAAATTCAAGCC | ATCAGTCCTTTGAAGCTGACCTTGC |
| *nos2b*-sgTarget-1 | ATTGCAGCACGTTATACTCTCTTAGC | AATGGGCACTGCTTGACTTTAT |
| *nos2b*-sgTarget-2 | ACTCACAAGAGCGAACTACTGC | ATTGACCCTTCACAAATCTTAGACG |
| *gsnor*-sgTarget-1 | ATTAATTTTCAGGTGATCAAATGTAAGGC | ATGGGGTGAAGTTGAGTCAGC |
| *gsnor*-sgTarget-2 | ATGTATTCAAAGTGTGTAATGTAGTCGG | TACCTGATTTTCTGGCACAGG |
