# Supplementary material for: cxcl18b-defined transitional state-specific nitric oxide drives injury-induced Müller glia cell-cycle re-entry in the zebrafish retina
Source: eLife. 2026 Jan 21;14:RP106274. doi: 10.7554/eLife.106274 (PMC12823065; doi:10.7554/eLife.106274)
Supplement: Supplementary file 2. [file elife-106274-supp2.docx]

**Supplementary File 2. Plasmid construction primer sequences, related to STAR Methods.**

| **Plasmid construction primer sequences** | | | |
| --- | --- | --- | --- |
| **Plasmid** | **Elements** | **Primer forward** | **Primer reverse** |
| *pTol2-cxcl18b: GFP* | *cxcl18b-promoter* | TAGGGCGAATTGGGCATTTGTCTCCTCATGCATTGACTAC | AATTGCTGCAAACTATATGTAGGAAATGCTG |
|  | *GFP* | ATGGTGAGCAAGGGC | TTACTTGTACAGCTCGTCCATGC |
| *pTol2-cxcl18b: gal4FF* | *gal4FF* | ATGAAGCTACTGTCTTCTATCGAACAAGC | CTACCCACCGTACTCGTCAATTCC |
| *pTol2-cxcl18b: Cre-vmhc: mCherry* | *Cre* | ATGGCCAATTTACTGACCGTACACC | CTAATCGCCATCTTCCAGCAGGC |
|  | *vmhc: mCherry* | CATGTGTCCTAAATTCTGAAAACACAGTAAGCC | TTACTTGTACAGCTCGTCCATG |
| *pTol2-UAS: Cas9-T2A-Cre-U6: nos2b sgRNA1; U6: nos2b sgRNA2* | *nos2b sgRNA1* | CTCGGGTCGCTCTTGTGAGTGACC | AAACGGTCACTCACAAGAGCGACC |
|  | *nos2b sgRNA2* | AGCTCGGGCTGGTGCCGCCAATGTCG | TAAACGACATTGGCGGCACCAGCCCG |
