# Supplementary material for: cxcl18b-defined transitional state-specific nitric oxide drives injury-induced Müller glia cell-cycle re-entry in the zebrafish retina
Source: eLife. 2026 Jan 21;14:RP106274. doi: 10.7554/eLife.106274 (PMC12823065; doi:10.7554/eLife.106274)
Supplement: Supplementary file 3. [file elife-106274-supp3.docx]

**Supplementary file 3. qPCR primer sequences, related to STAR Methods.**

| **Gene symbol** | **Primer forward** | **Primer reverse** |
| --- | --- | --- |
| *cxcl18b* | TGCTGCTCGCGGTAGTTTAC | TCTGCAGTAATTGGCCCTGC |
| *glula* | CGCATTACAGAGCCTGCCTA | ATTCCAGTTGCCTGGGATCG |
| *glulb* | ATGGTCCAGAACCAGCATCC | TCACAAGGGCCAACTTGGAA |
| *pcna* | CAGAAACCTAGCCATGGGGG | GTCTTGGACAGAGGAGTGGC |
| *nos1* | TGAGCACGTTTGCATAGGGT | CACTGGATTCGTCCCACACA |
| *nos2a* | CCAGATAACCACTGCTCTGCT | CTTGTCTTCCCATCTTGCTAAAA |
| *nos2b* | GAGGCAAAATTCCACCACCAG | TGCAAAGTGGTCATGGGACA |
| *gsnor* | ACAAGAAATCTCCACCCGGC | GACGGCTCGGATACTCTTCC |
| *β-actin* | ATTGCTGACAGGATGCAGAAG | GATGGTCCAGACTCATCGTACTC |
